# Correlation between growth differentiation factor 5 (rs143383) gene polymorphism and knee osteoarthritis: an updated systematic review and meta-analysis

**DOI:** 10.1186/s13018-021-02269-w

**Published:** 2021-02-19

**Authors:** Bin Jia, Yaping Jiang, Yingxing Xu, Yingzhen Wang, Tao Li

**Affiliations:** 1 Department of Joint Surgery, The Affiliated Hospital of Qingdao University, Qingdao, 266003 China; 2 Medical Department of Qingdao University, Qingdao, 266071 Shandong China; 3 Department of Oral Implantology, The Affiliated Hospital of Qingdao University, Qingdao, 266003 China

**Keywords:** Growth differentiation factor 5, Knee osteoarthritis, Genepolymorphism, Protection

## Abstract

**Background:**

A great deal of evidence has supported that growth differentiation factor 5 (GDF5) is associated with the occurrence of knee osteoarthritis (KOA), while their results are not consistent. In the present study, we aimed to explore the association between GDF5 gene polymorphism and KOA for a more credible conclusion.

**Methods:**

Comprehensive literature searches were carried out in English databases, including PubMed, Embase, Web of Science (WOS), and Cochrane, and Chinese databases, including China National Knowledge Infrastructure (CNKI), WANFANG, and VIP database. After the data were extracted from the required studies, the odds ratios (ORs) and their 95% confidence intervals (CIs) were determined to assess the correlation between GDF5 gene polymorphism and KOA. The publication bias was evaluated by funnel plot.

**Results:**

According to the inclusion and exclusion criteria, 15 studies on the correlation between GDF5 gene polymorphism and KOA occurrence were eligible for meta-analysis. Among these articles, four studies showed no apparent correlation, while the other 11 studies indicated an obvious correlation. Meanwhile, we also carried out a subgroup analysis of the population. Due to the inevitable heterogeneity, three genetic models were finally selected for analysis. With the allele model (C versus T: OR = 0.79, 95% CI = 0.73~0.87), recessive model (CC versus CT + TT: OR = 0.76, 95% CI = 0.68~0.86), and homozygous model (CC versus TT: OR = 0.66, 95% CI = 0.58~0.76), GDF5 gene polymorphism decreased the risk of KOA. Besides, a significant association was observed in Caucasians, Asians, and Africans. Meanwhile, the protective effect of genotype C (or CC) in the Asian group was little obvious than that in the Caucasian group and the African group. Although the quality of the included studies was above medium-quality, we obtained results with a low level of evidence.

**Conclusions:**

The results of the meta-analysis showed that the genotype C (or CC) of GDF5 protected against KOA occurrence in Caucasian, Asian, and African populations.

## Introduction

As the most common degenerative joint disease, osteoarthritis (OA) is the main factor of pain and disability in people aged over 45 years [1]. Although OA is common in the knee, it can also affect any other joints, including the hand and hip [2, 3]. The main pathogenesis of such disease involves irreversible destruction of cartilage, accompanied by the disrupted dynamic balance of chondrocytes, and the changes in other tissues [4]. However, the exact pathogenesis remains largely unclear, while it is believed that heredity greatly contributes to the pathogenesis of this disease [5].

Single nucleotide polymorphism (SNP), which accounts for more than 90% of human gene polymorphism, is the most common and stable gene variation in the human DNA chain [6]. As a member of the transforming growth factor β (TGF-β) superfamily, growth differentiation factor 5 (GDF5) plays a considerable role in the development, maintenance, and repair of cartilage and bone. Due to its important function, GDF5 is considered to be related to the OA [7].

A great deal of previous meta-analysis has supported that there is a correlation between GDF5 and knee osteoarthritis (KOA), while the research results remain contradictory. Some defects exist in the previous meta-analyses, such as the incorrect data extraction and the limitations of population subgroup analysis. On the other hand, there has been an update of the literature. In our present meta-analysis, we systematically and comprehensively evaluated the correlation between the GDF5 gene polymorphism and KOA occurrence.

## Materials and methods

### Literature retrieval

Based on the guidelines for the Preferred Reporting Item of Systematic Review and Meta-Analysis (PRISMA), a comprehensive literature search was conducted in English databases, including PubMed, Embase, Web of Science (WOS), and Cochrane, and Chinese databases, including China National Knowledge Infrastructure (CNKI), WANFANG, and VIP database (the latest literature was updated to July 13, 2020). We used a combination of medical subject heading terms (“GDF5” or “growth and differentiation factor 5” or “rs143383”) and (“polymorphism” or “SNP”) and (“osteoarthritis” or “OA”). Besides, references that could be included from the reviews and clinical trials were also manually searched.

### Inclusion and exclusion criteria

The inclusion criteria in this meta-analysis were set as follows: (1) human studies; (2) studies with a case-control group (case group: KOA subjects diagnosed by radiology; control group: subjects without the history of OA and autoimmune diseases); (3) studies on the relationship between GDF5 gene polymorphism and KOA susceptibility; and (4) studies with sufficient specific data to calculate odds ratios (ORs) and 95% confidence intervals (CIs). Meanwhile, the exclusion criteria were set as follows: animal model studies, reviews, case reports, expert opinions, and conference summaries. All the retrieved studies were screened by two reviewers according to the inclusion criteria and exclusion criteria.

### Data extraction

The following data were extracted from the included studies by two independent reviewers: the first author, the year of publication, the country and population of the subjects, the genotyping method, the number of alleles in the case group and control group, the sample size of the subjects in the case group and control group, and the *P* value for the Hardy–Weinberg equilibrium (HWE) test in the control group.

### Assessment of study quality

The Newcastle–Ottawa Scale (NOS) [8] was used to assess the quality of all studies based on the following three dimensions: selection (four items, 1 point each), comparability (one item, maximum 2 points), and exposure/outcome (three items, 1 point each). The score of each study ranged from 0 (worst) to 9 (best). The quality of each study was judged by two reviewers as low, medium, and high when a score of 0–3, 4–6, and 7–9 was obtained, respectively. If there was a difference in the scores given by the two reviewers, a consensus would be eventually reached through the discussion of each study.

### Statistical analysis

To clarify the relationship between GDF5 gene polymorphism and KOA susceptibility, the overall ORs and 95% CIs of the following five models were calculated: allele model (C versus T), dominant model (CC + CT versus TT), recessive model (CC versus CT + TT), heterozygous model (CT versus TT), and homozygous model (CC versus TT).

The heterogeneity test between studies was performed based on the *Q* statistics and *I*^2^ statistics of all studies in each model. If *P* < 0.1 and *I*^2^ ≥ 50%, it was considered that a large heterogeneity existed [9], and then the random-effects model was used. Otherwise, the fixed-effects model was chosen [10]. The source of heterogeneity was analyzed by subgroup analysis and sensitivity analysis by omitting each study in turn, and the publication bias was assessed using the funnel plot. The Review Manager 5.4 software (the Cochrane Collaboration, Oxford, UK) was used for the abovementioned analyses.

## Results

### Characteristics of the included studies

**Fig. 1:**
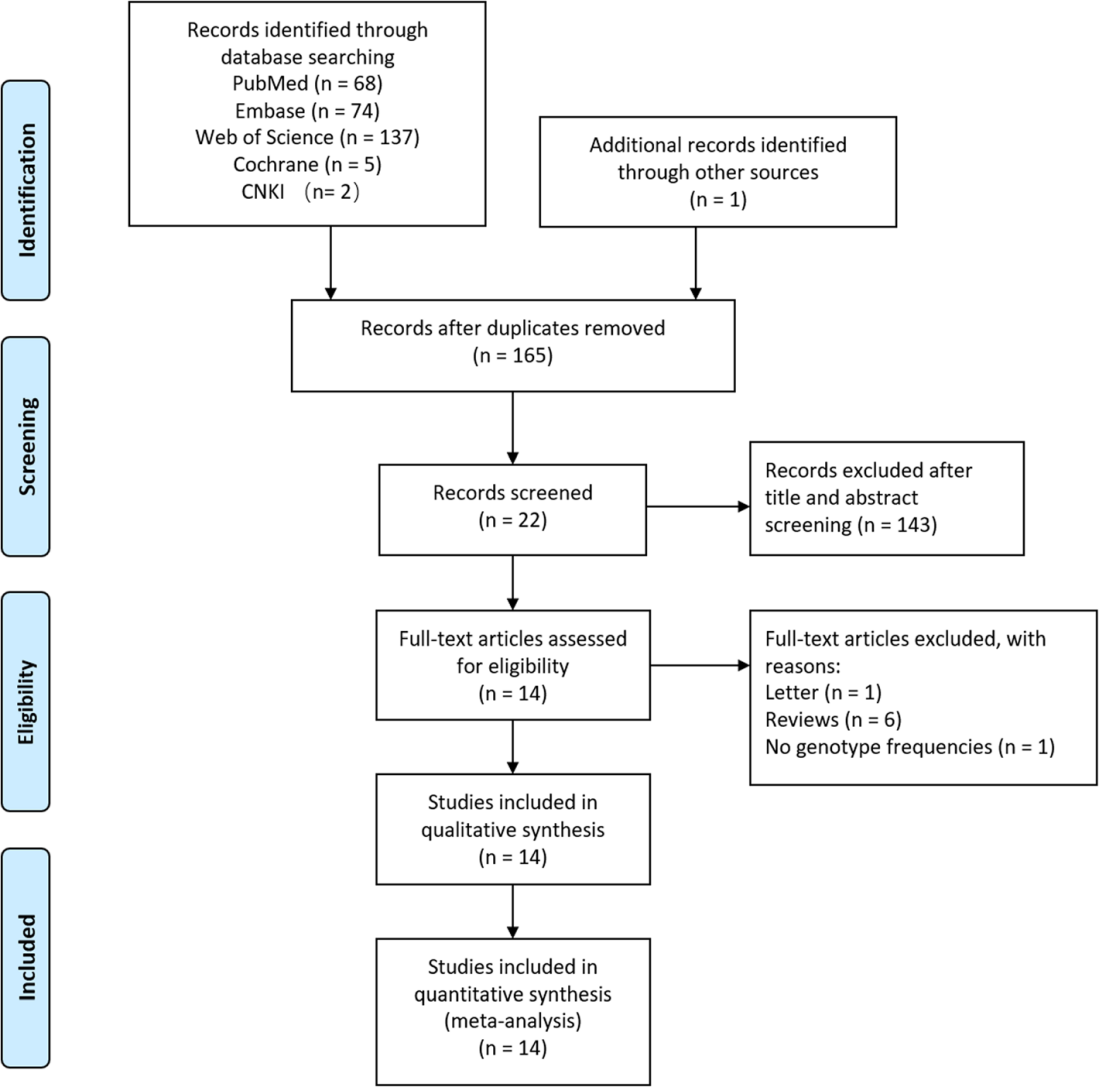
Flow chart of the study enrollment process

A total of 291 studies were retrieved from the following databases: PubMed (*n* = 68), Embase (*n* = 74), WOS (*n* = 137), Cochrane (*n* = 5), CNKI (*n* = 2), WANFANG (*n* = 5), and VIP database (*n* = 1), while one study was obtained by manual search. After removing repeated studies, and reading titles and abstracts, 22 studies were obtained. According to the established exclusion and inclusion criteria, eight articles were excluded (one letter, six reviews, and one article which could not extract allele frequency). Finally, 14 articles (15 studies) [11–24] consisting of 5524 KOA patients and 10,000 healthy controls were included in the meta-analysis (Fig. 1).

Tables 1 and 2 show the characteristics and quality of these 15 studies and gene frequency, including 13 high-score studies, and two medium-score studies. HWE test, which was used to analyze and evaluate the reliability of subjects’ choices in each study, indicated that the included studies were reliable.

**Table 1:** Characteristics of published studies for associations between GDF5 gene polymorphisms and KOA. Genotype and allelic distribution of GDF5 (C/T) gene polymorphisms among KOA patients and control individuals

Author, Year	Country	Population	Genotyping method	Case (KOA)/control (healthy)	Allele		Case			Allele		Control			HWE
					C	T	CC (%)	CT (%)	TT (%)	C	T	CC (%)	CT (%)	TT (%)	
**Miyamoto, 2007** [14]	Japan	Asian	TaqMan	718/861	305	1131	31 (4.32)	243 (33.84)	444 (61.84)	446	1276	58 (6.74)	330 (38.32)	473 (54.94)	0.965618
	China	Asian		313/485	135	491	19 (6.07)	97 (30.99)	197 (62.94)	289	681	48 (9.90)	193 (39.79)	244 (50.31)	0.28277
**Tsezou, 2008** [20]	Greece	Caucasian	DNA sequencer	251/267	186	316	30 (11.95)	126 (50.20)	95 (37.85)	213	323	44 (16.42)	125 (46.64)	99 (36.94)	0.668599
**Yao, 2008** **[**23**]**	China	Asian	RT-qPCR	313/485	135	491	19 (6.07)	97 (30.99)	197 (62.94)	289	681	48 (9.90)	193 (39.79)	244 (50.31)	0.28277
**Vaes, 2009** **[**21**]**	Netherland	Caucasian	TaqMan	667/2764	484	850	93 (13.94)	298 (44.68)	276 (41.38)	2160	3368	424 (15.34)	1312 (47.47)	1028 (37.19)	0.872858
**Valdes, 2009** **[**22**]**	UK	Caucasian	AS-PCR	259/509	168	350	35 (13.51)	98 (37.84)	126 (48.65)	412	606	84 (16.50)	244 (47.94)	181 (35.56)	0.907919
**Takahashi, 2010** [18]	Japan	Asian	TaqMan	933/1225	421	1445	54 (5.79)	313 (33.55)	566 (60.66)	621	1829	80 (6.53)	461 (37.63)	684 (55.84)	0.844647
**Cao, 2010** **[**11**]**	Korea	Asian	PCR-RFLP	276/298	137	415	11 (3.99)	115 (41.68)	150 (54.35)	165	431	26 (8.72)	113 (37.92)	159 (53.36)	0.3605
**Tawonsawatruk, 2011** **[**19**]**	Thailand	Asian	PCR-RFLP	90/103	63	117	11 (12.22)	41 (45.56)	38 (42.22)	93	113	23 (22.33)	47 (45.63)	33 (32.04)	0.424487
**Shin, 2012** [17]	Korea	Asian	HRMA	725/1737	381	1069	38 (5.24)	305 (42.07)	382 (52.69)	901	2573	106 (6.10)	689 (39.67)	942 (54.23)	0.17575
**Mishra, 2017** **[**13**]**	India	Asian	PCR-RFLP	500/500	376	624	75 (15.00)	226 (45.20)	199 (39.80)	466	534	97 (19.40)	272 (54.40)	131 (26.20)	0. 37456
**Ozcan, 2017** **[**16**]**	Turkey	Caucasian	PCR-RFLP	94/279	71	117	14 (14.89)	43 (45.74)	37 (39.36)	257	301	52 (18.64)	153 (54.84)	74 (26.52)	0.083439
**Elazeem, 2017** **[**12**]**	Egypt	African	TaqMan	50/50	44	56	14 (28.00)	16 (32.00)	20 (40.00)	51	49	13 (26.00)	25 (50.00)	12 (24.00)	0.997742
**Zhang, 2019** **[**24**]**	China	Asian	PCR-RFLP	288/397	223	353	59 (20.49)	105 (36.45)	124 (43.06)	223	571	32 (8.06)	159 (40.05)	206 (52.39)	0.864938
**Mohasseb, 2019** **[**15**]**	Egypt	African	PCR-RFLP	47/40	43	51	10 (21.28)	23 (48.94)	14 (29.78)	35	45	11 (27.50)	13 (32.50)	16 (40.00)	0. 31687

*KOA* knee osteoarthritis, *HWE* Hardy–Weinberg equilibrium

**Table 2:** Quality assessment of case-control studies

Author, Year	Is the case definition adequate?	Representativeness of the cases	Selection of controls	Definition of controls	Comparability of cases and controls on the basis of the design or analysis	Ascertainment of exposure	Same method of ascertainment for cases and controls	Non-response rate	NOS
**Miyamoto, 2007 (1)** [14]	⭐	⭐	⭐	⭐	NA	⭐	⭐	NA	6
**Miyamoto, 2007 (2)** [14]	⭐	⭐	⭐	⭐	NA	⭐	⭐	NA	6
**Tsezou, 2008** [20]	⭐	⭐	⭐	⭐	⭐	⭐	⭐	NA	7
**Yao, 2008** **[**23**]**	⭐	⭐	⭐	⭐	⭐⭐	⭐	⭐	NA	8
**Vaes, 2009** **[**21**]**	⭐	⭐	⭐	⭐	⭐⭐	⭐	⭐	NA	8
**Valdes, 2009** **[**22**]**	⭐	⭐	⭐	⭐	⭐	⭐	⭐	NA	7
**Takahashi, 2010** [18]	⭐	⭐	⭐	⭐	⭐	⭐	⭐	NA	7
**Cao, 2010** **[**11**]**	NA	⭐	⭐	⭐	⭐⭐	⭐	⭐	Na	7
**Tawonsawatruk, 2011** **[**19**]**	⭐	⭐	⭐	⭐	⭐⭐	⭐	⭐	NA	8
**Shin, 2012** [17]	⭐	⭐	⭐	⭐	⭐⭐	⭐	⭐	NA	8
**Mishra, 2017** **[**13**]**	⭐	⭐	⭐	⭐	⭐⭐	⭐	⭐	NA	8
**Ozcan, 2017** **[**16**]**	⭐	⭐	⭐	⭐	⭐	⭐	⭐	NA	7
**Elazeem, 2017** **[**12**]**	⭐	⭐	⭐	⭐	⭐⭐	⭐	⭐	NA	8
**Zhang, 2019** **[**24**]**	NA	⭐	⭐	⭐	⭐⭐	⭐	⭐	NA	7
**Mohasseb, 2019** **[**15**]**	⭐	⭐	⭐	⭐	⭐⭐	⭐	⭐	NA	8

⭐ = 1 score; ⭐⭐ = 2 scores; *NOS* Newcastle–Ottawa Scale

### Meta-analysis results

During the meta-analysis, we found that there was large heterogeneity in all five genetic models. The random-effects model was selected for analysis, and the source of heterogeneity was further analyzed. The aggregate data of all studies showed that genotype C (or CC) in the GDF5 gene polymorphism had a significant protective effect against KOA. Table 3 presents the details: allele model (C versus T: OR = 0.83, 95% CI = 0.74~0.93, *P*<0.00001), dominant model (CC + CT versus TT: OR = 0.78, 95% CI = 0.67~0.90, *P*<0.00001), recessive model (CC versus CT + TT: OR = 0.80, 95% CI = 0.65~0.99, *P* = 0.0002), heterozygous model (CT versus TT: OR = 0.79, 95% CI = 0.69~0.91, *P*<0.0001), and homozygous model (CC versus TT: OR = 0.70, 95% CI = 0.55~0.90, *P*<0.00001).

**Table 3:** Evaluation of the association between GDF5 gene polymorphisms and KOA susceptibility

		Origin			Final		
Comparison	Group	OR (95% CI)	Heterogeneity test		OR (95% CI)	Heterogeneity test	
			*P*	*I*^2^		*P*	*I*^2^
**Allele model (C versus T)**	Overall	0.83 [0.74, 0.93]	<0.00001	77%	0.79 [0.73, 0.87]	0.009	54%
	Caucasian	0.82 [0.72, 0.93]	0.23	30%	0.82 [0.72, 0.93]	0.23	30%
	Asian	0.83 [0.70, 0.99]	<0.00001	86%	0.78 [0.68, 0.88]	0.002	69%
	African	0.89 [0.59, 1.34]	0.39	0%	0.89 [0.59, 1.34]	0.39	0%
**Dominant model (CC + CT versus TT)**	Overall	0.78 [0.67, 0.90]	<0.00001	74%	NA		
	Caucasian	0.74 [0.58, 0.94]	0.06	59%			
	Asian	0.79 [0.65, 0.96]	<0.00001	81%			
	African	0.86 [0.26, 2.78]	0.06	72%			
**Recessive model (CC versus CT + TT)**	Overall	0.80 [0.65, 0.99]	0.0002	65%	0.76 [0.68, 0.86]	0.73	0%
	Caucasian	0.83 [0.69, 1.00]	0.80	0%	0.83 [0.69, 1.00]	0.80	0%
	Asian	0. 78 [0.55, 1.10]	<0.00001	79%	0.71 [0.60, 0.83]	0.52	0%
	African	0.91 [0.47, 1.76]	0.51	0%	0.91 [0.47, 1.76]	0.51	0%
**Heterozygous model (CT versus TT)**	Overall	0.79 [0.69, 0.91]	<0.0001	68%	NA		
	Caucasian	0.75 [0.58, 0.98]	0.05	62%			
	Asian	0.80 [0.68, 0.96]	0.0003	73%			
	African	0.88 [0.17, 4.46]	0.02	82%			
**Homozygous model (CC versus TT)**	Overall	0.70 [0.55, 0.90]	<0.00001	72%	0.66 [0.58, 0.76]	0.39	6%
	Caucasian	0.73 [0.60, 0.89]	0.54	0%	0.73 [0.60, 0.89]	0.54	0%
	Asian	0.69 [0.47, 1.03]	<0.00001	83%	0.61 [0.50, 0.74]	0.22	26%
	African	0.81 [0.38, 1.72]	0.54	0%	0.81 [0.38, 1.72]	0.54	0%

### Subgroup analysis and heterogeneity analysis

**Fig. 2:**
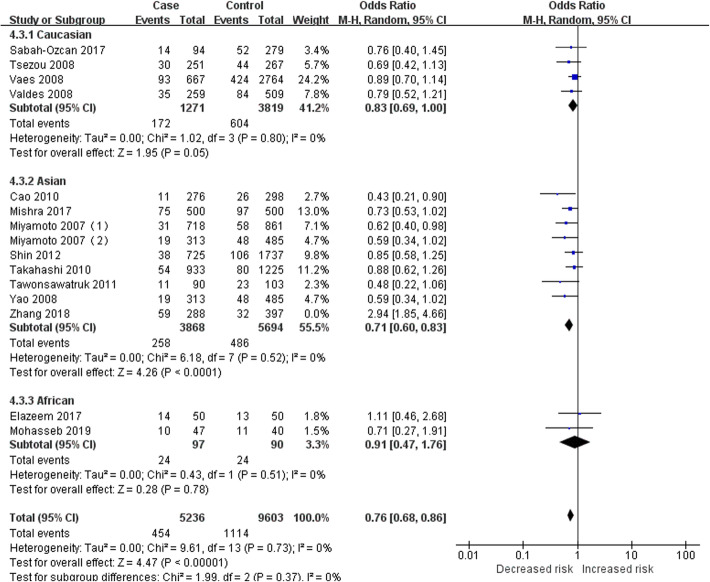
Forest plot of the correlation between GDF5 gene polymorphism and KOA risk. Recessive model (CC versus CT + TT)

**Fig. 3:**
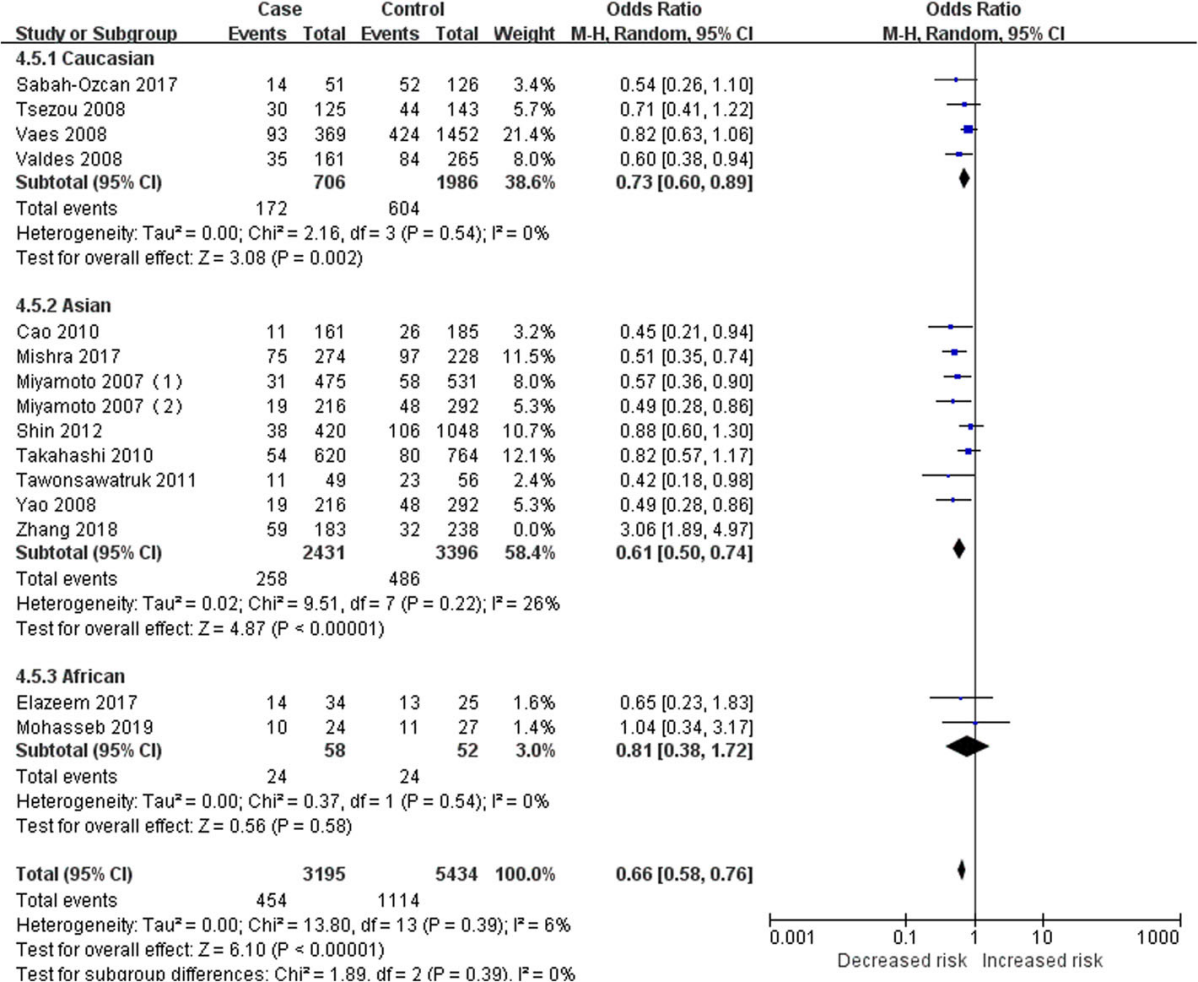
Forest plot of the correlation between GDF5 gene polymorphism and KOA risk. Homozygous model (CC versus TT)

**Fig. 4:**
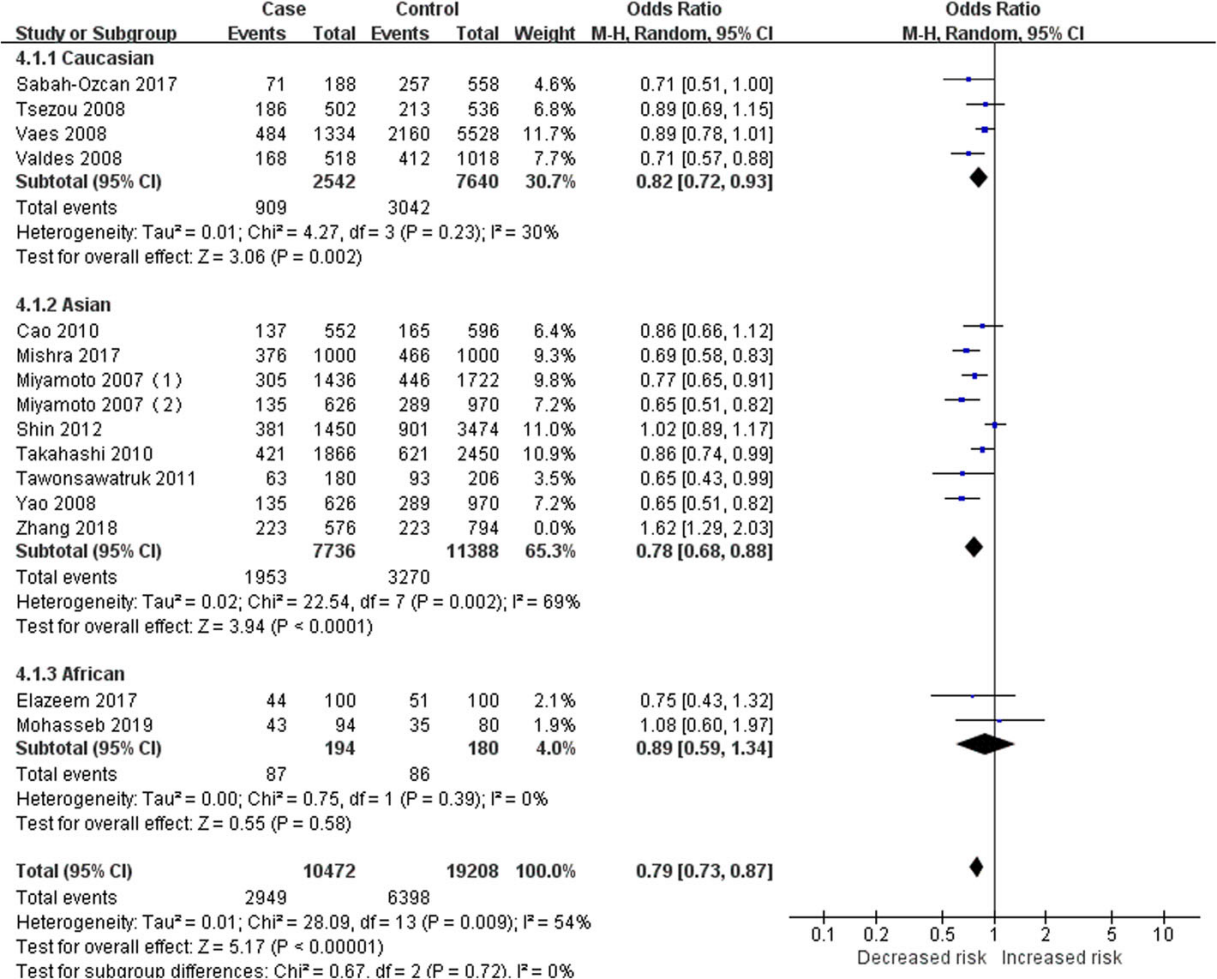
Forest plot of the correlation between GDF5 gene polymorphism and KOA risk. Allele model (C versus T)

To identify the source of heterogeneity, the subgroup analysis was performed, since previous studies have shown different results in various populations [1–24]. Three subgroups, Caucasian, Asian, and African groups, were included in this analysis according to the population of the subjects. Table 3 presents the results of subgroup analysis that genotype C (or CC) in the GDF5 gene polymorphism still had a significant protective effect against KOA in the Caucasian group, Asian group, and African group. The *I*^2^ > 50% in the subgroups of the dominant model and heterozygous model could not reduce the heterogeneity by excluding each study. Consequently, these two genetic models were not suitable for the evaluation of the correlation between GDF5 gene polymorphism and KOA. In the other three genetic models, the heterogeneity of the Caucasian group (allele model: *P* = 0.23, *I*^2^ = 30%; recessive model: *P* = 0.80, *I*^2^ = 0%; homozygous model: *P* = 0.54, *I*^2^ = 0%) and African group (allele model: *P* = 0.39, *I*^2^ = 0%; recessive model: *P* = 0.51, *I*^2^ = 0%; homozygous model: *P* = 0.54, *I*^2^ = 0%) was low, which could even be 0%, while that of the Asian group (allele model: *P*<0.00001, *I*^2^ = 86%; recessive model: *P*<0.00001, *I*^2^ = 79%; homozygous model: *P*<0.00001, *I*^2^ = 83%) was relatively high. The study of the Asian group could be inferred as the source of heterogeneity. After the studies of Shin et al. [17] and Zhang et al. [24] were excluded from the allele model, the heterogeneity of the subgroup and the population was significantly decreased (Asian group: *P* = 0.18, *I*^2^ = 32%; overall: *P* = 0.15, *I*^2^ = 30%). After the study of Zhang et al. [24] was excluded, the heterogeneity was decreased in the recessive model (Asian group: *P* = 0.52, *I*^2^ = 0%; overall: *P* = 0.73, *I*^2^ = 0%. Fig. 2) and homozygous model (Asian group: *P* = 0.22, *I*^2^ = 26%; overall: *P* = 0.39, *I*^2^ = 6%. Fig. 3). Moreover, we carefully analyzed the study of Zhang et al. [24] and found that the OR and 95% CI calculated based on the data provided in this article were not consistent with the final results in the original text. We thought that unreliable data might be the source of heterogeneity. However, after analyzing the study of Shin et al. [17], we did not find anything that could explain the heterogeneity. Therefore, the study of Shin et al. [17] could not be eliminated. Heterogeneity in the allele model was hardly changed (Asian group: *P* = 0.002, *I*^2^ = 69%; overall: *P* = 0.009, *I*^2^ = 54%. Fig. 4).

### Sensitivity analysis

Compared with the original results, there was no obvious difference between the results of sensitivity analysis and the original results, suggesting that the overall results were stable (method: omitting each study in turn).

### Publication bias

**Fig. 5:**
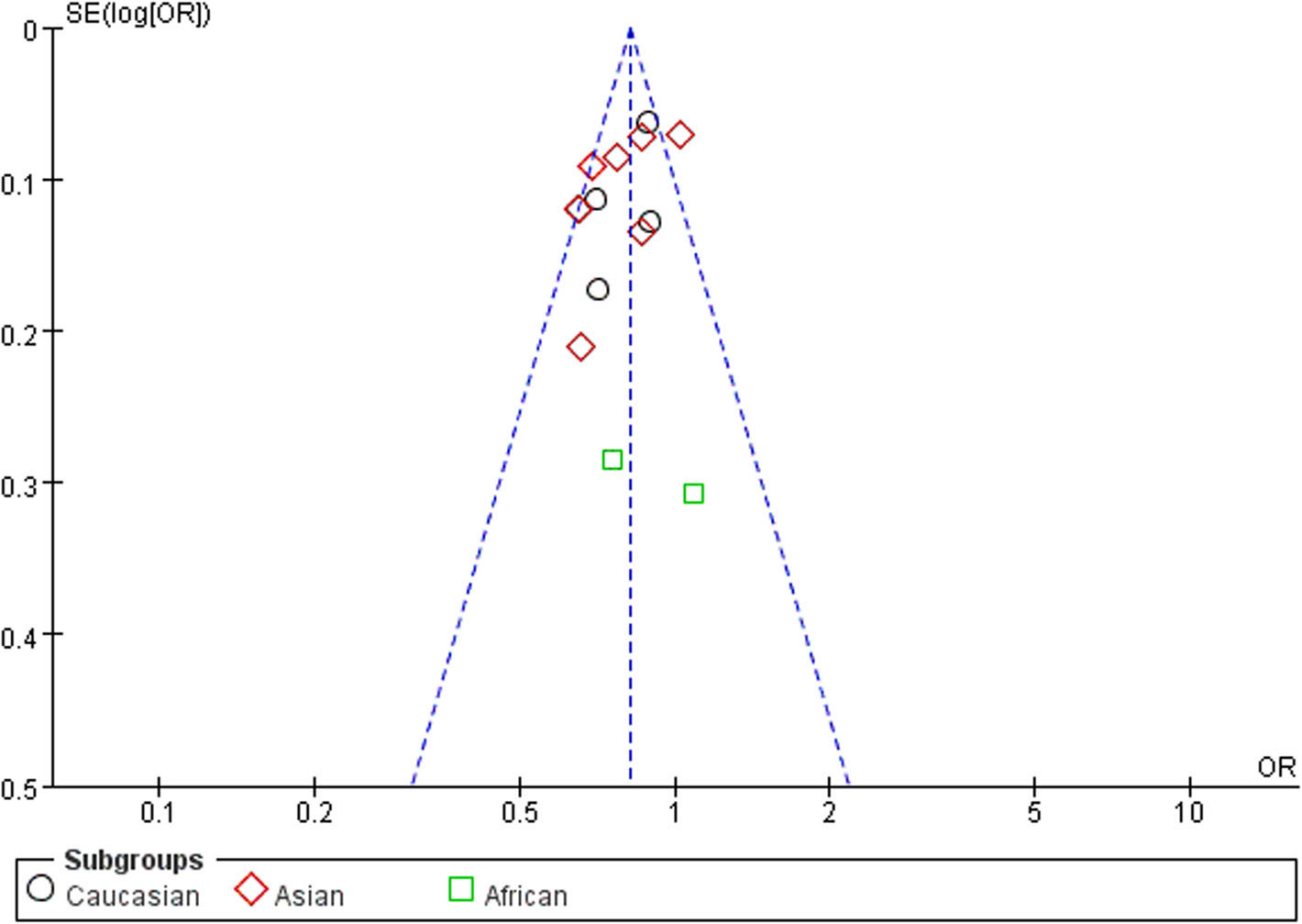
Funnel plot for publication bias among selected studies. Allele mode (C versus T)

**Fig. 6:**
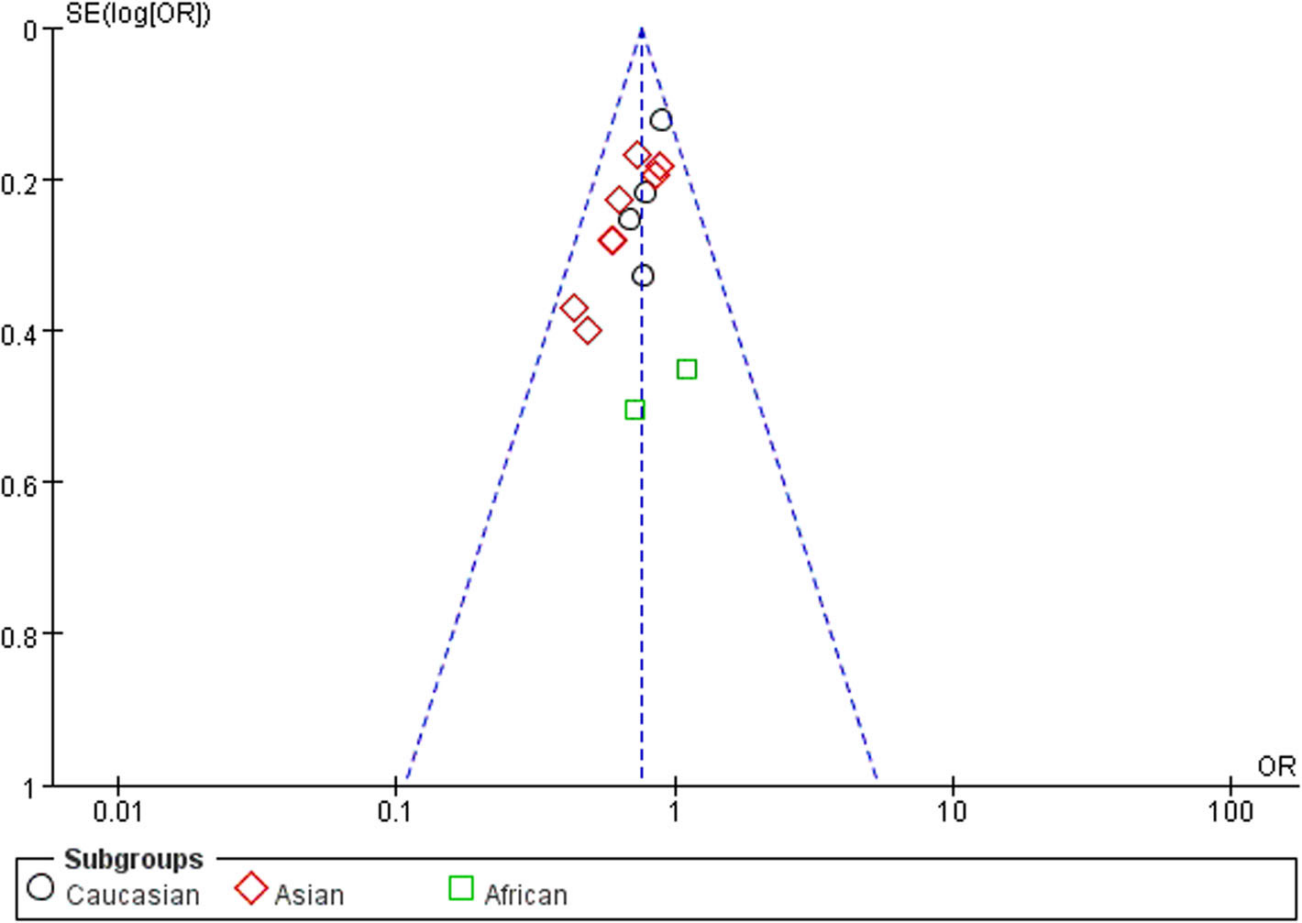
Funnel plot for publication bias among selected studies. Recessive model (CC versus CT + TT)

**Fig. 7:**
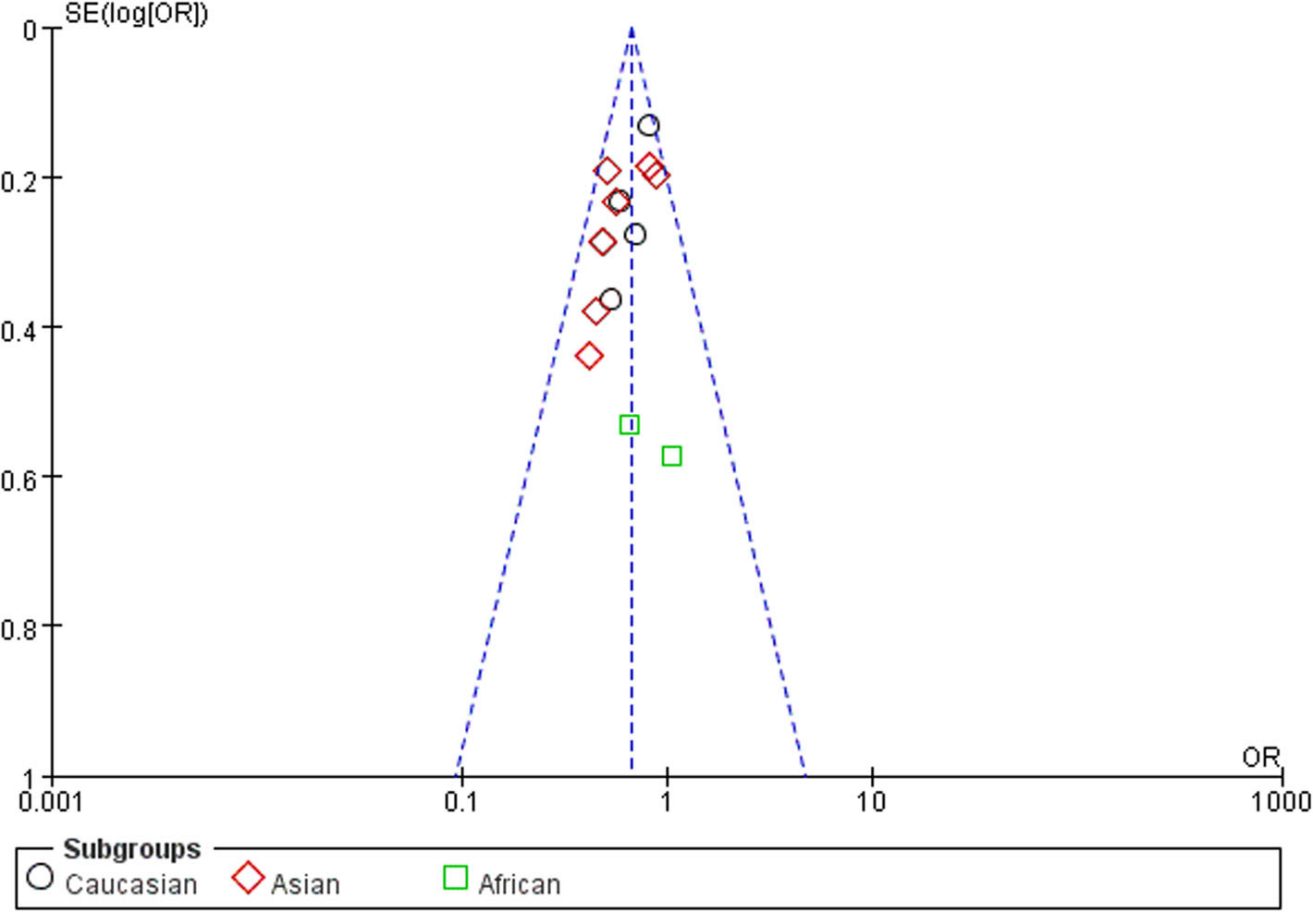
Funnel plot for publication bias among selected studies. Homozygous model (CC versus TT)

In order to assess the publication bias of the literature, funnel plots, Egger’s test, and Begg’s test were performed. The funnel plots indicated that there was no obvious publication bias (Figs. 5, 6, and 7). Meanwhile, the Egger’s test was performed to provide the statistical evidence (allele model: *P* = 0.386, recessive model: *P* = 0.776, and homozygous model: *P* = 0.356).

### GRADE evidence evaluation

This study contains a total of three genetic model analyses. The quality of evidence for each analysis result is low (Table 4).

**Table 4:** GRADE evidence evaluation

Outcomes	Illustrative comparative risks (95% CI)		Relative effect (95% CI)	No of Participants (studies)	Quality of the evidence (GRADE)
	Assumed risk	Corresponding risk			
	Control	SNP			
**C versus T**	**Study population**		**OR 0.78** (0.72 to 0.84)	24,756 (13 studies)	⊕ ⊕ ⊝ ⊝ **Low**
	**349 per 1000**	**295 per 1000** (279 to 311)			
	**Moderate**				
	**391 per 1000**	**334 per 1000** (316 to 350)			
**CC versus CT + TT**	**Study population**		**OR 0.76** (0.68 to 0.86)	14,839 (14 studies)	⊕ ⊕ ⊝ ⊝ **Low**
	**116 per 1000**	**91 per 1000** (82 to 101)			
	**Moderate**				
	**153 per 1000**	**121 per 1000** (109 to 134)			
**CC versus TT**	**Study population**		**OR 0.66** (0.58 to 0.76)	8629 (14 studies)	⊕ ⊕ ⊝ ⊝ **Low**
	**205 per 1000**	**145 per 1000** (130 to 164)			
	**Moderate**				
	**292 per 1000**	**214 per 1000** (193 to 239)			

## Discussion

As a common crippling disease, OA has a great impact on patients and society [25, 26]. Among all types of OA, KOA gives the most burden for people [27]. Up to date, there is no particularly effective way to cure KOA except for total knee arthroplasty. Although OA is considered to be a multifactorial disease, it has been reported that genetic factors play an important role in the pathogenesis of the disease [28]. Previous studies have shown the correlation between GDF5 (rs143383) gene polymorphism and KOA. However, the conclusions of these different studies are not consistent. The studies of Cao et al. [11], Shin et al. [17], Takahashi et al. [18], and Tsezou et al. [20] have indicated that there is no obvious correlation between the GDF5 gene polymorphism and KOA. Therefore, we aimed to explore the correlation between GDF5 gene polymorphism and KOA in this meta-analysis.

In recent years, a great deal of attention has been paid to the GDF5 gene. GDF5, a member of the bone morphogenetic protein (BMP) family, is involved in a variety of cellular processes related to bone repairs, such as proliferation, differentiation, and angiogenesis, as well as bone and cartilage formation [29]. Like other BMPs, GDF5 can initiate its signal cascade by binding to transmembrane serine/threonine kinase I and type II receptors. The binding of GDF5 leads to the phosphorylation of the receptor, which activates the downstream Smad signaling pathway, and then Smads shift to the nucleus to regulate the transcription of various genes [30–32]. Another pathway is that both GDF5 and BMP2 bind to type I receptors, and the recruitment of type II receptors by the ternary complex causes the polymer complex to trigger the MAPK pathway [33]. These are examples of how GDF5 works. Mutations in genes can lead to the loss of their original function or even the adverse effect. Therefore, it seems to be a good idea to prevent KOA in advance by studying the correlation between the GDF5 gene polymorphism and KOA occurrence.

In our present meta-analysis, we abandoned the dominant model and heterozygous model because of the irreducible heterogeneity. In the remaining three genetic models, the analysis of overall studies, Caucasian group, Asian group, and African group showed that the GDF5 gene polymorphism was significantly associated with the susceptibility to KOA, suggesting that genotype C (or CC) had a protective effect against KOA. However, in the studies of Cao et al. [11], Shin et al. [17], Takahashi et al. [18], and Tsezou et al. [20], there is no obvious correlation between the GDF5 gene polymorphism and KOA. After the included studies were merged, the results became meaningful among the Caucasian group, Asian group, and African group. Furthermore, we found that the protective effect of genotype C (or CC) in the Asian group was slightly more obvious compared with the Caucasian group and African group. However, the differences among the subgroups were not significant (Table 3 and Figs. 2, 3, and 4). This finding suggested that the difference in population had little effect on the correlation between the GDF5 gene polymorphism and KOA. As far as we know, there have been some meta-analyses of GDF5 and KOA, such as the recent study by Kazem et al. [34]. After studying these works, we found that minor mistakes existed in the data extraction of some studies, such as the study of Miyamoto et al. [14]. Besides, the subgroup analysis of the previous meta-analysis is only done in the Caucasian group and Asian group. Therefore, we included the African population data in our meta-analysis, although there were only two studies. The protective function provided by genotype C (or CC) of GDF5 was also observed in the African group. The GRADE evidence quality evaluation system was used by us to evaluate the results of the analysis, which was not available in other meta-analyses.

Nevertheless, there are some defects in the present analysis. First, the language was restricted to English and Chinese, which might limit the research population and lead to bias. Secondly, there was no more stratified analysis of factors (including gender, BMI, and so on). Although the included studies are all medium- or high-quality studies, the subject of this meta-analysis is different from traditional case-control studies, which made upgrade non-existent. According to the GRADE methodology quality evaluation, the analysis results of the three genetic models are all at low levels of evidence. Further research may have an important impact on the confidence interval of the effect size and may change the effect size. We still need to wait for more well-designed case-control studies to be added to the analysis.

## Conclusions

Collectively, our current meta-analysis suggested that GDF5 gene polymorphism was associated with KOA susceptibility. In the three genetic models (allele model, recessive model, and homozygous model), genotype C (or CC) of GDF5 had a protective effect against KOA in Caucasian, Asian, and African populations.

## Data Availability

All data generated or analyzed during this study are included in this published article and its supplementary information files.

## Abbreviations

GDF5: Growth differentiation factor 5
KOA: Knee osteoarthritis
WOS: Web of Science
CNKI: China National Knowledge Infrastructure
CIs: Confidence intervals
OA: Osteoarthritis
SNP: Single nucleotide polymorphism
TGF-β: Transforming growth factor β
PRISMA: Preferred Reporting Item of Systematic Review and Meta-Analysis
HWE: Hardy–Weinberg equilibrium
NOS: Newcastle–Ottawa Scale
BMPs: Bone morphogenetic proteins
